# Alkaline salt impairs germination recovery more severely than neutral salt in the euhalophyte *Suaeda salsa*

**DOI:** 10.3389/fpls.2026.1937990

**Published:** 2026-10-07

**Authors:** Xiao Liu

**Affiliations:** 1 School of Geography and Tourism, Qilu Normal University, Jinan, China; 2 Shandong Jinan Urban Ecosystem National Observation and Research Station, Qilu Normal University, Jinan, China

**Keywords:** enforced dormancy, halophyte, osmotic adjustment, physiological drought, recovery potential

## Abstract

**Introduction:**

Soil salinization and alkalization increasingly threaten global ecosystem stability, necessitating a clearer understanding of how dominant halophytes respond to complex salt stresses.

**Methods:**

This study examined the differential effects of neutral and alkaline salt stresses on seed germination and early seedling growth of *Suaeda salsa*, a dominant halophyte in the Yellow River Delta. A two-factor factorial experiment was conducted using three salt types (NaCl, KCl, NaHCO_3_) across six concentration gradients (0–500 mmol L^-1^). A two-stage germination trial, comprising an initial stress phase and a post-stress recovery phase, assessed root and shoot growth, fresh mass accumulation, water potential, and germination ratios.

**Results:**

Salt concentration primarily determined germination traits. At comparable molar concentrations, alkaline salt (NaHCO_3_) exerted significantly stronger inhibition on growth than the neutral salts (NaCl, KCl). Low-concentration NaCl promoted fresh mass accumulation and sustained shoot elongation through efficient osmotic adjustment, whereas alkaline stress severely impaired this capacity, inducing acute physiological drought. The secondary germination trial revealed an “enforced dormancy” strategy: the effects of neutral salt stress were largely reversible, with markedly enhanced germination upon stress relief, whereas alkaline salt stress reduced recovery potential, fundamentally compromising recovery potential.

**Discussion:**

Neutral and alkaline components must be distinguished when evaluating soil degradation. Future ecological restoration in the Yellow River Delta should prioritize amelioration of high-pH alkaline conditions to ensure natural regeneration of halophyte communities.

## 1 Introduction

The combination of climate change and human activity has significantly accelerated soil salinization, transforming it into a formidable challenge for modern ecosystems (Guo et al., 2023). The detrimental impacts of soil salinization are extensive and multi-dimensional. Initially, the elevated salt concentrations in the soil increase the osmotic pressure of the soil solution, which in turn hinders the growth and the ability of plant roots to absorb water and nutrients (Parvin et al., 2020). This results in poor root development and compromised plant health (Paul et al., 2024). Salinization accelerates soil water evaporation, thereby inducing soil drought and moisture depletion. Concurrently, the hygroscopic nature of soil salts restricts plant access to available moisture, severely impeding water uptake for growth (El-Ghannam et al., 2023). Excessively high salt concentrations can inhibit normal physiological processes within plants, affecting photosynthesis and respiration, ultimately leading to plant death (Yang et al., 2011). Salinization affects the growth and activity of soil microorganisms, disrupting material cycling and nutrient transformation processes in soil (Wei et al., 2024). For ecosystems, salinization significantly reduces plant diversity, as most plant species are unable to survive and reproduce in high-salt environments (Xu et al., 2024). In addition, soil salinization alters the biochemical processes in the soil, affecting the structure and function of microbial communities, which impacts the material cycling and energy flow of the entire ecosystem (Nordio et al., 2024). Salinization also leads to soil compaction and structural damage, reducing soil aeration and water infiltration capacity, further exacerbating the degradation process of ecosystems (Mesele et al., 2025).

Seeds and seedlings within the regeneration niche are indispensable for maintaining community productivity and stabilizing ecosystem structure (Wang et al., 2023). However, the escalating severity of land salinization and alkalization presents a significant challenge to the growth of these plant components. Saline-alkali soils are characterized by the presence of various ions, such as Na^+^, K^+^, Cl^–^, and CO_3_^2–^, typically forming a complex mixture of different salts. Neutral salt stress primarily inhibits plant growth through osmotic effects, whereas alkaline salt stress, distinguished by a high pH, can exert more severe detrimental effects (Wu et al., 2025). Consequently, neutral and alkaline salt stresses represent two distinct forms of stress, to which plants exhibit different adaptive mechanisms. Salt stress markedly reduces seed germination ratio, root length, and shoot length, thereby prolonging the germination period, particularly under high salt concentrations (Alemán et al., 2025). Thus, understanding the influence of varying salt types and concentrations on seed germination is crucial for enhancing the utilization of saline-alkali land and preserving ecosystem stability.

Precipitation events can provide temporary alleviation from salt stress (Park et al., 2024). The subsequent recovery of seed germination following this alleviation is a complex process that involves the coordinated interplay of multiple physiological and molecular mechanisms. Although seeds subjected to NaCl stress exhibit significantly reduced germination ratio, root lengths, and shoot lengths, they retain a certain capacity for recovery once the stress is relieved. This recovery potential may be linked to osmolytes and antioxidant enzyme activities accumulated by the seeds during the stress period, which facilitate their rapid regrowth once salt levels decrease (Debez et al., 2018). However, the diverse salt compositions found in saline-alkali soils introduce uncertainty regarding plants’ recovery potential post-stress relief (Sun et al., 2022). Therefore, alongside understanding how different types and concentrations of salt stress impact seed germination, investigating the recovery potential of plants after relief from varying salt stress conditions is also crucial.

Approximately 50% of the land in the Yellow River Delta region is characterized by saline-sodic soil, rendering it a typical area for studying such conditions. Soil salinization in this region poses a significant challenge to local ecological balance, exhibiting distinct spatial heterogeneity ranging from mild to severe. To align the experimental design with realistic ecological conditions, the selection of salt types and concentration gradients was directly based on field investigations of the region’s soil properties. NaCl was the dominant neutral salt responsible for local salinization, while NaHCO_3_ was the primary alkaline salt driving the elevation of soil sodicity. Furthermore, the concentration gradient of 0–500 mmol L^–1^ employed in this study was used to simulate the trends in the salinity gradient of the natural habitat of *Suaeda salsa* (L.) Pall. *S. salsa*, a widely distributed halophyte in the Yellow River Delta, mitigates cellular toxicity by sequestering absorbed salt ions into vacuoles and accumulating organic osmolytes to balance osmotic potential. Concurrently, it dilutes internal salt concentrations through leaf succulence, relying on these physiological and morphological adaptations to tolerate high-salinity environments. Although the stronger inhibition by alkaline than neutral salts has been repeatedly documented, this evidence derives mainly from glycophytic crops and forage species (Yang et al., 2011; Wu et al., 2025; Zhu et al., 2024). Whether the same pattern holds in a euhalophyte such as *S. salsa* is not self-evident: because *S. salsa* actively absorbs Na^+^ and uses it for osmotic adjustment, the Na^+^ delivered by NaHCO_3_ could, in theory, be exploited by the plant and partially offset the high-pH damage. A two-factor factorial experiment was therefore set up to explore the response strategies of *S. salsa* under different salt types and concentrations, and its recovery after salt stress relief; the inclusion of KCl as a third salt additionally separated the cation effect (NaCl *vs* KCl) from the alkalinity effect (NaCl *vs* NaHCO_3_). It was hypothesized that (1) alkaline salt stress exerts more pronounced effects than neutral salt stress even in this Na^+^-specialized euhalophyte, i.e., the high-pH component overrides the potential benefit of the accompanying Na^+^; and (2) the effects of neutral salt stress may be reversible, whereas those of alkaline salt stress may be largely irreversible.

## 2 Materials and methods

### 2.1 Experimental design

The seed germination experiment was conducted at Qilu Normal University in Jinan City, Shandong Province, China (36.66° N, 117.50° E). Seeds of *S. salsa* were collected from the *S. salsa* community in the Yellow River Delta (38.14° N, 118.71° E) in late October, 2024, and stored in a low-temperature storage cabinet at 4 °C. Before the experiment, the 2,3,5-triphenyltetrazolium chloride (TTC) staining method was used to assess seed viability. Seed viability was 98.7% (987 of 1000 seeds stained red). Healthy, plump, uniform, brown seeds were selected for the seed germination experiment, which was conducted from April 15^th^ to May 4^th^, 2025, in an incubator. The seeds were surface-sterilized with 2% (v/v) NaClO for 10 min and rinsed three times with distilled water. To break dormancy, they were then soaked in distilled water at 4 °C for 24 h. The experiment was conducted at 25°C, 2000 lx light intensity in the daytime (04:00–20:00) and 0 lx light intensity at night (20:00–04:00); the light was supplied by fluorescent lamps. Fifty seeds were evenly placed in one Petri dish with filter paper for germination. The seed germination experiment was separated into two stages.

#### 2.1.1 Initial germination under salt stress

Salt types (T) and salt concentrations (C) were selected in the two-factor factorial experiment. Three salt types were chosen: T1, NaCl; T2, NaHCO_3_; T3, KCl. Six salt concentrations were designed: C1, 0 mmol L^–1^; C2, 50 mmol L^–1^; C3, 100 mmol L^–1^; C4, 200 mmol L^–1^; C5, 300 mmol L^–1^; C6, 500 mmol L^–1^. The solution pH was measured by high precision pH test paper (pH 5.5–9.0, d = 0.5), the pH of NaCl and KCl solution was 7.0 while that of NaHCO_3_ solution was 8.5. Eighteen treatments (T*i*C*j*; *i* = 1–3, *j* = 1–6) were designed; each treatment had four replications (four Petri dishes). A total of 72 Petri dishes were used for the experiment. The initial germination (GR1, [Equation (1)]) experiment was concluded after three consecutive days without germination. Ten seedlings in each Petri dish were randomly selected for growth traits and fresh mass measurements, the data for each replication were the average of the ten chosen seedlings. All germinated seeds in each Petri dish were used for water potential measurement.

#### 2.1.2 Secondary germination after salt stress relief

After initial germination, all germinated seeds were removed, and the rest were used for the secondary germination. Then, all ungerminated seeds and Petri dishes were carefully rinsed with distilled water to relieve salt stress, and then the seeds were put back in their original Petri dishes with filter paper and distilled water for germination. The secondary germination (GR2, [Equation (2)]) experiment was concluded after three consecutive days without germination.

### 2.2 Root and shoot growth traits

Root and shoot growth traits include root length (RL, mm), shoot length (SL, mm), and root-to-shoot length ratio (R:S). The solution on the surface of the ten selected seedlings was blotted with filter paper. Then, root length and shoot length were measured with a ruler (d = 1 mm). Root-to-shoot length ratio was calculated as the root length divided by the shoot length.

### 2.3 Fresh mass and water potential

After root and shoot growth trait measurements, the selected seedlings were weighed for fresh mass (FM, g). Then, all germinated seeds in each Petri dish were used for seedling water potential (Ψ, MPa) measurement. After surface moisture was blotted with filter paper, seedling water potential was measured by a dewpoint hygrometer (WP4C, METER Group, Pullman, WA, USA), and the temperature in the sample room was set at 25.0°C. The solution water potential (Π, MPa) was measured by the same dewpoint hygrometer with the temperature in the sample room set at 25.0°C.

### 2.4 Germination ratio

Germination ratio was calculated as the number of germinated seeds divided by the number of seeds. The germination ratio was calculated for each dish and for each of the two stages separately:

(1) $$\text{GR}1=\frac{\text{G}1}{50},$$

(2) $$\text{GR}2=\frac{\text{G}2}{50-\text{G}1},$$

(3) $$\text{GRT}=\frac{\text{G}1+\text{G}2}{50},$$

where GR1, GR2, GRT were the initial germination ratio, secondary germination ratio, total germination ratio ([Equation (3)]), respectively; G1 and G2 were the number of germinated seeds in the initial germination and secondary germination, respectively.

### 2.5 Statistics

Data were checked for normality (Shapiro-Wilk test) and homogeneity (Levene test). Two-way analysis of variance (two-way ANOVA) followed by Duncan’s multiple comparison test was used to test the main effect and interaction of salt type and salt concentration on growth traits, seedling water potential, and germination ratio. One-way ANOVA followed by Duncan’s multiple comparison test was used to detect the differences among concentrations within the same type. One-sample *t*-test was used to detect the differences between seedling water potential and solution water potential. The critical value *P* was set at 0.05. All statistical analyses were performed using the IBM SPSS Statistics 26 software package (IBM Co., Amonk, NY, USA), and all figures were drawn using OriginPro 2024 SR1 (OriginLab Co., Northampton, MA, USA).

## 3 Results

### 3.1 Effects of salt type and salt concentration on germination traits

Variations in germination traits were predominantly explained by salt type and concentration. Salt concentration explained the largest share of variation for growth traits, seedling water potential and the initial germination ratio (*η*^2^ = 0.542–0.998), whereas for the secondary and total germination ratios the interaction dominated (*η*^2^ = 0.471 and 0.389) (**Table 1**).

**Table 1:** The proportion of explained variation (*η*^2^) of salt type (T), salt concentration (C), and their interaction on germination traits.

Germination traits	Type	Concentration	T × C	Total *η*^2^
Root length	0.121**	0.826**	0.052**	0.999
Shoot length	0.155**	0.775**	0.061**	0.991
Root-to-shoot length ratio	0.150**	0.802**	0.039**	0.991
Fresh mass	0.359**	0.542**	0.096**	0.997
Seedling water potential	0.000	0.998**	0.000	0.998
Initial germination ratio	0.000	0.908**	0.017	0.925
Secondary germination ratio	0.104**	0.165**	0.471**	0.740
Total germination ratio	0.150**	0.342**	0.389**	0.881

Data stand for the Type III sum of square of factors and their interaction (SSi), divided by the total sum of square (SSt), SSi/SSt. **, *P* < 0.01.

### 3.2 Root and shoot growth traits

The effect on root and shoot growth traits varied significantly among salt types (**Table 1**). At the same concentration, the inhibitory effect of KCl was greater than that of NaCl, and lower than that of NaHCO_3_ (**Figure 1**).

**Figure 1:**
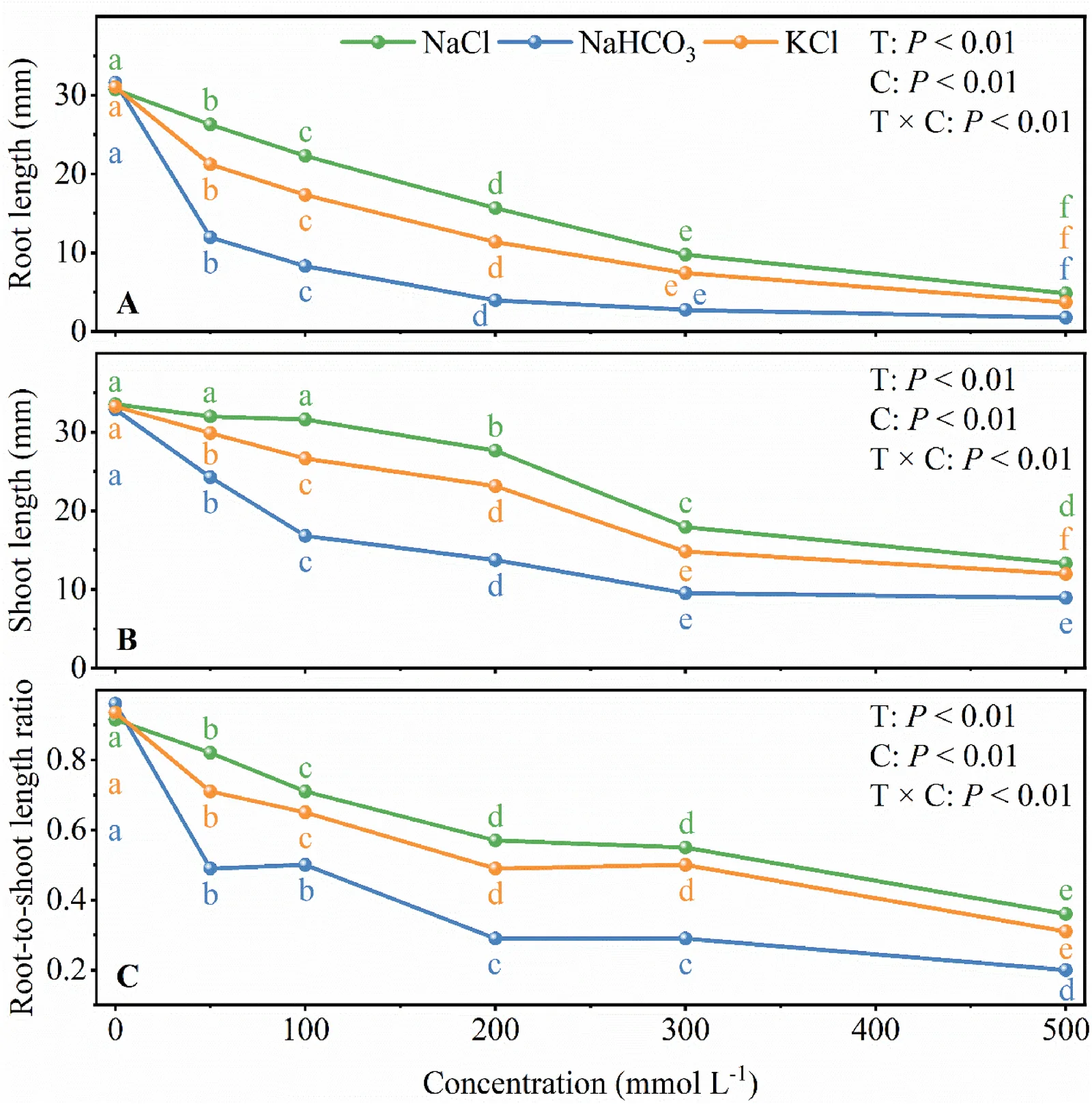
Root length **(A)**, shoot length **(B)**, and root-to-shoot length ratio **(C)** of *S. salsa* under salt stress in different types (T) and concentrations (C). Two-way ANOVA was used to detect the main effect and interaction of type and concentration, one-way ANOVA was used to detect the differences among concentrations within the same type, *n* = 4. Different letters indicate significant differences among concentrations within the same type, *P* < 0.05.

With increasing salt concentrations:

1. Root length decreased significantly (**Figure 1A**).
2. Shoot length significantly shortened in the NaHCO_3_ group and the KCl group; in the NaCl group, when salt concentration was less than 100 mmol L^–1^, shoot length had no obvious difference among treatments; when salt concentration was more than 100 mmol L^–1^, shoot length significantly shortened (**Figure 1B**).
3. Root-to-shoot length ratio significantly decreased; however, it was relatively stable when salt concentration was between 100 and 200 mmol L^–1^; in the NaHCO_3_ group, the relatively stable state also appeared when salt concentration was between 50 and 100 mmol L^–1^ (**Figure 1C**).

### 3.3 Fresh mass and water potential

Effects of salt type and salt concentration were significant on fresh mass, while seedling water potential was mostly dependent on salt concentration (**Table 1**). At the same concentration, the effect of KCl on fresh mass was larger than that of NaCl, and lower than that of NaHCO_3_ (**Figures 2A–C**).

**Figure 2:**
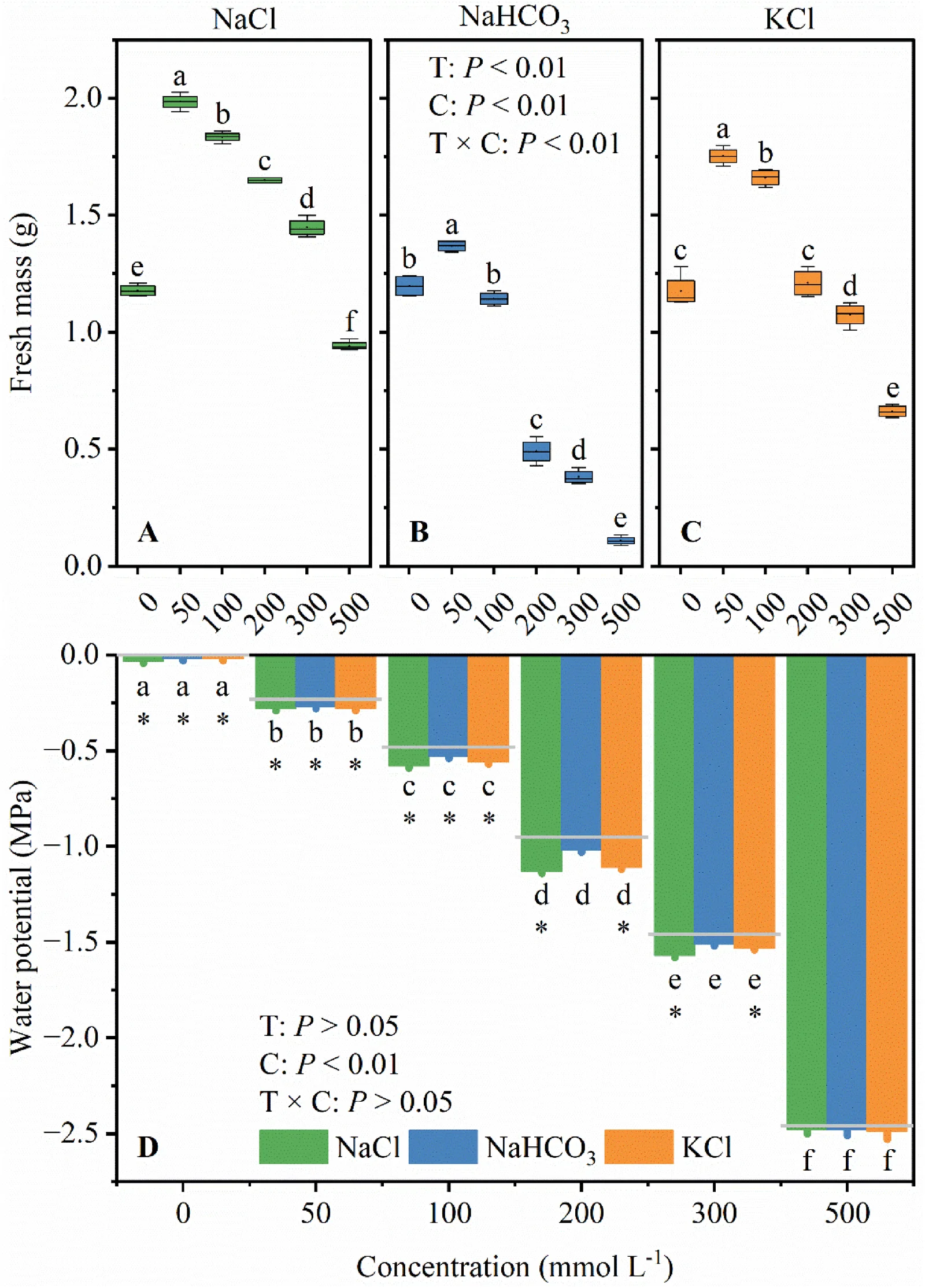
Fresh mass (**A**, NaCl; **B**, NaHCO_3_; **C**, KCl) and seedling water potential **(D)** of *S. salsa* under salt stress in different types and concentrations. The gray lines in D represent the solution water potential in different treatments. Two-way ANOVA was used to detect the main effect and interaction of type and concentration. One-way ANOVA was used to detect the differences among concentrations within the same type, different letters indicate significant differences among concentrations within the same type, *n* = 4, *P* < 0.05. One-sample *t*-test was used to detect the differences between seedling water potential and solution water potential, *n* = 4, *, *P* < 0.05.

As salt concentration gradually increased from 50 to 500 mmol L^–1^, fresh mass significantly decreased. Moreover, in the NaCl group, fresh mass at 0 mmol L^–1^ was significantly lower than that at 300 mmol L^–1^, but higher than that at 500 mmol L^–1^ (**Figure 2A**); in the NaHCO_3_ group, fresh mass at no salt stress had no obvious difference from that at 100 mmol L^–1^ (**Figure 2B**); in the KCl group, fresh mass at no salt stress had no obvious difference from that at 200 mmol L^–1^ (**Figure 2C**).

As salt concentration gradually increased, seedling water potential and solution water potential significantly decreased. Under NaCl and KCl stress, significant differences between seedling and solution water potentials were observed from 0 to 300 mmol L^–1^. Under NaHCO_3_ stress, this difference was significant between 0 to 100 mmol L^–1^ (**Figure 2D**).

### 3.4 Germination ratio

The initial germination ratio was higher when salt concentration was from 50 to 100 mmol L^–1^ (**Figure 3**). The total germination ratio was higher than the initial germination ratio. After salt stress relief, the recovery potential of the KCl group (63.50, **Figure 3C**) was higher than that of the NaHCO_3_ group (48.50, **Figure 3B**), while it was lower than that of the NaCl group (92.25, **Figure 3A**). As the original salt concentration increased, in the NaCl group, the secondary germination ratio gradually increased; in the NaHCO_3_ and KCl groups, the secondary germination ratio reached its highest value at 50 mmol L^–1^ and 100 mmol L^–1^ (**Figure 3D**). Moreover, when the concentration was higher than 200 mmol L^–1^, the secondary germination ratio of the KCl group was significantly higher than that of the NaHCO_3_ group, while it was significantly lower than that of the NaCl group (**Figure 3D**).

**Figure 3:**
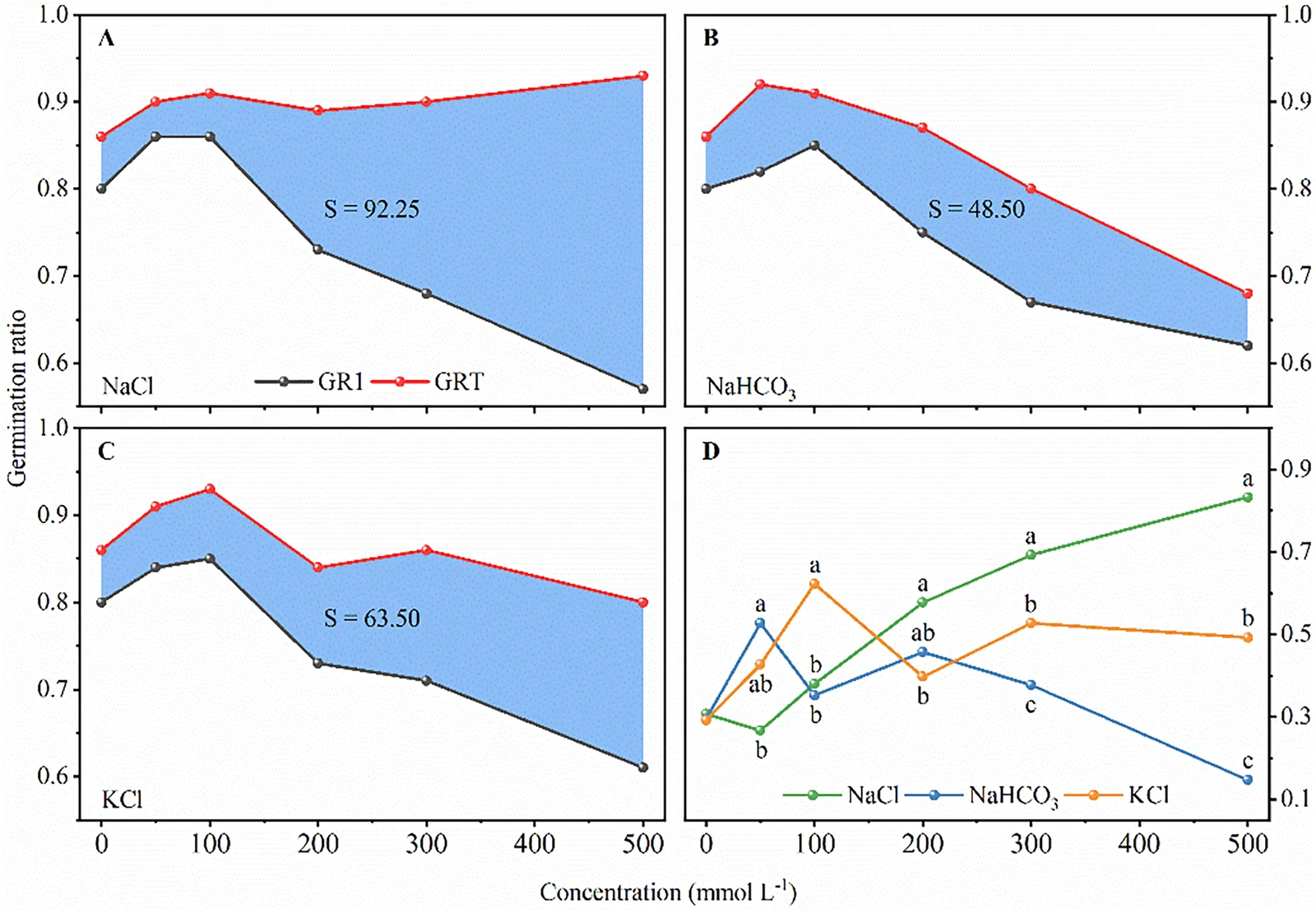
Initial germination ratio (GR1), total germination ratio (GRT) of *S. salsa* under salt stress in different types (**A**, NaCl; **B**, NaHCO_3_; **C**, KCl) and concentrations, and secondary germination ratio of *S. salsa* **(D)**. Different letters stand for significance among salt types under the same concentration, *n* = 4, *P* < 0.05. Blue zone represents the difference between GR1 and GRT, the number is the area of the zone.

## 4 Discussion

### 4.1 Alkaline salt can exhibit stronger inhibition than neutral salt

The results of this study indicate that salt stress significantly inhibited the growth of *S. salsa* seedlings. As depicted in **Figure 1**, root length, shoot length, and the root-to-shoot length ratio all exhibited a gradually declining trend with increasing salt concentrations. This overall growth inhibition is consistent with the well-documented effects of elevated external salt concentrations on plant water relations and ion balance. Under salt stress, the degree of inhibition on root length was significantly greater than that on shoot length, indicating that the root system exhibits higher stress sensitivity than the aboveground parts. This shift in resource allocation strategy may represent an adaptive regulation by plants under adverse conditions (El-Ghannam et al., 2023), whereby aboveground fresh mass is prioritized to maintain photosynthetic capacity while root elongation is sacrificed under osmotic constraint. Among the neutral salts, the inhibitory effect of Na^+^ was weaker than that of K^+^. One plausible explanation for this pattern is that, owing to long-term adaptation to Na^+^-rich environments, *S. salsa* may possess a relatively greater capacity to cope with Na^+^, whereas excessive extracellular K^+^ could impose additional ionic stress to which the species is less well adapted. However, because we did not directly measure Na^+^ or K^+^ accumulation, tissue Na^+^/K^+^ ratios, or ion transport processes, this interpretation should be regarded as a possible mechanism rather than a demonstrated physiological response. Future studies incorporating measurements of ion accumulation and transport are needed to test this hypothesis.

Additionally, significant differences were observed in the inhibitory effects of different salt types on growth; the inhibitory effect of alkaline salts was stronger than that of neutral salts (**Figure 1**). The elevated pH associated with NaHCO_3_ can alter rhizosphere conditions and potentially impair the plant’s ability to maintain normal water and nutrient uptake; accordingly, the stronger inhibition by NaHCO_3_ than by the neutral salts should be interpreted as the coupled osmotic–pH stress of alkaline salinization rather than as the effect of a single isolated factor. The high pH environment of NaHCO_3_ led to a more pronounced inhibition of root elongation compared to shoot growth, resulting in a significantly reduced root-to-shoot ratio under alkaline stress, reflecting a fundamental hindrance to root system development. This suggests that alkaline stress imposes constraints beyond simple osmotic effects, potentially interfering with mineral ion acquisition and overall metabolic function. Therefore, alkaline salts exert a more severe inhibition on seed germination compared to neutral salts (Zhang et al., 2024). Regarding neutral salts, the inhibitory effect of Na^+^ was weaker than that of K^+^. As a euhalophyte, *S. salsa* has evolved adaptive traits to utilize Na^+^ in osmotic adjustment; conversely, an abnormally high external K^+^ concentration may disturb the ionic balance that the plant is accustomed to maintaining, resulting in relatively stronger growth inhibition.

It should be noted that, because the treatments were established on a molar-concentration basis, the three salts at the same nominal concentration differ in actual osmotic potential and pH; the salt-type effects reported here therefore represent the integrated stress of each salt type rather than isolated single-factor effects. Notably, the contrast between the two neutral salts is minimally confounded, as NaCl and KCl share similar dissociation behavior and near-neutral pH, whereas the stronger inhibition by NaHCO_3_ should be interpreted as the coupled osmotic–pH stress of alkaline sodic conditions under field-relevant scenarios. Future studies adopting isosmotic or pH-controlled designs would help strictly disentangle these two components.

### 4.2 Low salt stress may promote fresh mass via osmotic adjustment

With increasing salt concentrations, the fresh mass of *S. salsa* seedlings presented a trend of initially increasing and then decreasing. Specifically, in the NaCl treatment group, the fresh mass of seedlings under 50–300 mmol L^-1^ stress was even significantly higher than that of the control (**Figure 2A**). This profoundly indicates the unique adaptive mechanism of *S. salsa*. At moderate salt concentrations, the plant may actively absorb inorganic ions such as Na^+^ to lower its cellular osmotic potential, thereby promoting water absorption and accumulation, which ultimately manifests as an increase in fresh mass (Yang et al., 2011). Fresh mass is widely regarded as a reliable trait for salt-tolerance evaluation; the ion-driven osmotic adjustment underlying its increase is a hallmark of euhalophytes, characterized by succulence achieved through the accumulation of inorganic ions, and reflects a sophisticated ecological adaptation that enables them to colonize and dominate in saline habitats where glycophytes cannot survive. Understanding these adaptive mechanisms in halophytes is not only crucial for ecological stability but also holds significant economic relevance for the utilization of salt-tolerant plant resources (Garcia-Caparros et al., 2023; An et al., 2025). However, when the salt concentration continued to rise beyond the plant’s tolerance threshold, as indicated in **Figures 2B, C**, fresh mass significantly decreased, as excessive osmotic stress became the dominant factor inhibiting plant growth and fresh mass accumulation (Pandey et al., 2018). The inhibitory effect of alkaline salts on seedling fresh mass was stronger than that of neutral salts, manifested by the inhibition of fresh mass increase at lower NaHCO_3_ concentrations, which may be attributed to the combined effects of high pH and osmotic stress on the plant’s water uptake capacity (Yang et al., 2011). Consistent with the growth traits, the inhibitory effect of K^+^ on fresh mass was also stronger than that of Na^+^, likely due to the species’ relatively lower tolerance to excessive K^+^ compared with Na^+^ (Meng et al., 2019). It is important to note that dry mass was not determined in this study. Given that the seedlings were extremely small, ensuring the measurement accuracy of dry mass was technically difficult despite the availability of numerous seedlings. This constitutes a limitation of the present study, and future investigations should incorporate dry mass measurements to provide a more comprehensive assessment of fresh mass accumulation patterns.

Changes in water potential further revealed the physiological strategies of *S. salsa* under different salt stresses. As salt concentration increased, both solution water potential and seedling water potential decreased significantly (**Figure 2D**). It is noteworthy that the relationship between seedling water potential and external solution water potential differed significantly under different salt types, directly reflecting the strength of the plant’s osmotic adjustment capability. Under NaCl and KCl treatments, when the concentration did not exceed 500 mmol L^-1^, seedling water potential was significantly lower than the solution water potential. This indicates that *S. salsa* maintains a steeper water potential gradient relative to the external environment within this concentration range. This gradient facilitates water uptake and sustains turgor pressure under stress, reflecting the species’ efficient osmotic adjustment as a halophyte (Parvin et al., 2020). Under NaHCO_3_ treatment, **Figure 2D** reveals that seedling water potential was largely consistent with solution water potential, with a significant difference detected between 0 to 100 mmol L^–1^. This result strongly demonstrates that alkaline salt stress severely weakens the plant’s osmotic adjustment capacity. Consequently, the plant cannot effectively lower its internal water potential to sustain a favorable water-uptake gradient, resulting in severe physiological drought (Zhu et al., 2024). This also explains, from a whole-plant water relations perspective, why fresh mass accumulation and growth under alkaline salt stress were more severely inhibited. The species-specific molecular basis underlying these adaptive responses has been partially elucidated in closely related Suaeda species (Khramov et al., 2024), providing a framework for future investigations into the differential tolerance observed here.

### 4.3 Seed germination may suffer largely irreversible damage under alkaline salt stress

In the initial salt stress stage, the germination ratio of *S. salsa* exhibited a trend of “initially increasing and then decreasing” with increasing salt concentration (**Figure 3**). At low-to-medium salt concentrations (50–100 mmol L^-1^), the germination ratio was significantly higher than that of other treatments, revealing the unique ecological adaptability of this species as a halophyte. A moderate saline environment may help maintain intracellular ion homeostasis or act as compatible osmolytes to promote seed water uptake, thereby breaking seed dormancy or initiating metabolism (Gu et al., 2024). Comparative studies across halophytic and non-halophytic species have further demonstrated that the ability to maintain germination capacity under moderate salinity is a defining ecological trait of salt-tolerant flora. However, with further increases in salt concentration, the dual effects of high osmotic potential and ion toxicity severely inhibited seed germination, leading to a significant decrease in germination ratio, which is consistent with the findings of most studies on the effects of salt stress on seed germination (Parvin et al., 2020). The results of the secondary germination experiment after stress relief clearly reflected the differentiated impacts of different salt types on seed recovery potential (**Figure 3D**). This study found that the total germination ratio of all treatment groups after stress relief was higher than the initial germination ratio. For instance, the recovery potential of the KCl group was higher than that of the NaHCO_3_ group, while it was lower than that of the NaCl group, indicating that some seeds did not die during the stress period but entered a state of “enforced dormancy”. This strategy enables seeds to preserve viability in harsh environments and quickly resume germination when environmental conditions improve, serving as an important mechanism for plants to adapt to fluctuating environments (Mohamed et al., 2023). Seed germination and the subsequent restoration of root vitality upon stress relief are critical processes that facilitate this rapid recovery (Habibi et al., 2025).

Specifically, this post-stress recovery response induces a state of physiological readiness by accumulating osmolytes and activating antioxidant enzyme systems during the initial stress phase. This pre-activation allows seeds to mount faster defense responses and enhances their tolerance upon stress relief, thereby facilitating rapid water uptake and germination recovery (Debez et al., 2018). However, significant differences existed in the recovery potential across salt types: recovery after NaCl stress relief was the best, whereas recovery after NaHCO_3_ stress relief was the worst. This difference strongly supports the hypothesis proposed in this study, namely that the effects of neutral salt stress are reversible, whereas those of alkaline salt stress are largely irreversible. As a neutral salt, NaCl mainly inhibits germination through osmotic stress; when external salts are washed away, the osmotic barrier is eliminated, and seeds can rapidly resume water uptake and germinate. Specifically, as shown in **Figure 3A**, in the NaCl group, the secondary germination ratio gradually increased with the rise of initial salt concentration, indicating that high concentrations of NaCl may have induced deeper enforced dormancy while retaining higher recovery potential (Cai et al., 2022). In contrast, the lowest recovery rate was observed in the NaHCO_3_ group (**Figure 3B**), as NaHCO_3_ stress is accompanied by a high pH environment, causing more severe physiological damage to seeds. Consequently, even upon stress relief, these seeds fail to resume germination, yielding the lowest recovery rate observed. In summary, *S. salsa* seeds exhibited robust tolerance and recovery potential under neutral salt stress, whereas sodic conditions posed a more severe and largely irreversible challenge to population regeneration.

## 5 Conclusion

This study systematically evaluates the differential impacts of neutral and alkaline salt stresses on the seed germination and early seedling growth of *S. salsa*, a dominant halophyte in the Yellow River Delta. The findings validate the initial hypothesis that alkaline stress exerts more profound detrimental effects than neutral salt stress. Specifically, while low-to-moderate concentrations of NaCl promoted fresh mass accumulation and maintained shoot elongation through a highly efficient osmotic adjustment mechanism, NaHCO_3_ stress severely impaired this regulatory capacity, leading to physiological drought and growth inhibition. Furthermore, the two-stage germination experiment strongly supports the second hypothesis regarding the reversibility of stress effects. The impacts of neutral salt stress were predominantly reversible, as evidenced by the significantly enhanced secondary germination ratio following stress relief, which indicates an effective “enforced dormancy” strategy. Conversely, alkaline salt stress caused persistent inhibition after stress relief, resulting in a substantially compromised recovery potential. These findings highlight the critical necessity of distinguishing between neutral and alkaline components when assessing soil salinization. From an ecological perspective, the robust resilience and specific adaptive mechanisms of *S. salsa* under neutral salt stress underscore its vital role in maintaining ecosystem stability. However, the increasing alkalization of saline soils poses a severe, potentially irreversible threat to population regeneration. Therefore, future ecological restoration efforts in the Yellow River Delta should prioritize the amelioration of high-pH alkaline conditions to better support the natural regeneration of halophyte communities.

## Data Availability

The original contributions presented in the study are included in the article/**Supplementary Material**. Further inquiries can be directed to the corresponding author.
